# Supplementary material for: Direct digital sensing of protein biomarkers in solution
Source: Nat Commun. 2023 Feb 6;14:653. doi: 10.1038/s41467-023-35792-x (PMC9902533; doi:10.1038/s41467-023-35792-x)
Supplement: Supplementary file 3 — Description of Additional Supplementary Files [file 41467_2023_35792_MOESM3_ESM.pdf]

Title: Supplementary Software

Description: Computer code used in this article for the analysis of photon time traces. Code is available in a single compressed zip file ("digitISA.zip"). The folder contains the custom code (burstLoc.py, getBursts_nature.py, Leefilter.py, and readPTU.py) and a README.txt file with instructions on how to use the code. A demo data set is also provided in the subfolder "DEMO", which contains raw data for the time trace shown in Figure 2c (right panel, red). The source code was written in Python (version 3.7). A description of the code's functionality is provided in Methods (Data analysis). Code is also available on the GitHub repository: https://github.com/rj380cam/digitISA/. The software's license for use is GNU Lesser General Public License v2.1; a license file ("LICENSE") is provided.
